# STINGing organelle surface with acid

**DOI:** 10.1038/s44319-024-00120-x

**Published:** 2024-03-19

**Authors:** Yoshihiko Kuchitsu, Tomohiko Taguchi

**Affiliations:** Laboratory of Organelle Pathophysiology, Department of Integrative Life Sciences, Graduate School of Life Sciences, Tohoku University, Sendai, Japan

**Keywords:** Autophagy & Cell Death, Membranes & Trafficking

## Abstract

This article reflects on recent findings on the ion channel function of STING in the context of "conjugation of ATG8 (LC3) to single membranes (CASM)".

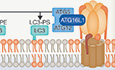

## Teaser

The cyclic GMP-AMP synthase (cGAS)-stimulator of interferon genes (STING) innate immune pathway has emerged as a critical driver of inflammation in a variety of settings, such as virus infection, cellular stress, tissue damage, and aging. The pathway detects microbial and host-derived double-stranded DNA (dsDNA) in the cytosol and triggers the production of type I interferons and proinflammatory cytokines that help eliminate the invading pathogens. STING is a mobile protein. After its binding to cyclic GMP-AMP, which is generated by cGAS in the presence of cytosolic dsDNA, STING exits the ER, translocates to the Golgi and then to recycling endosomes (REs), finally reaching lysosomes. In the course of its travel, STING recruits TBK1 from the cytosol and triggers type I interferon and proinflammatory responses through the activation of IRF3 and NF-κB at the trans-Golgi network (TGN).

**Figure 1:**
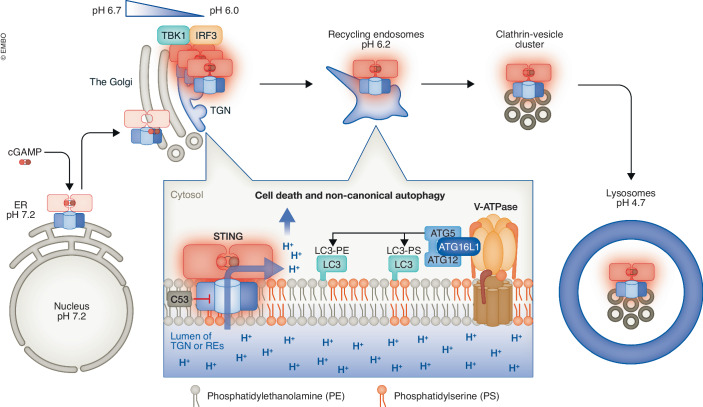
STING regulates non-canonical autophagy through its proton channel activity. As a dimer localized to the ER, STING undergoes translocation to the Golgi upon binding its ligand cyclic GMP-AMP (cGAMP). At the TGN, a subdomain of the Golgi, STING clusters and activates innate immune signaling through activation of TBK1/IRF3. Subsequently, STING translocates to recycling endosomes, and is ultimately engulfed by lysosomes through microautophagy. The luminal pH at the TGN and recycling endosomes is mildly acidic resulting in proton efflux to the cytosol through the pore in the STING dimer. De-acidification of these organelles activates the v-ATPase/ATG16L1 axis to conjugate phosphatidylethanolamine (PE) and phosphatidylserine (PS) with LC3.

Intriguingly, STING also induces the lipidation of STING-associated membranes with microtubule-associated protein 1 light chain 3 (LC3) (Saitoh et al, 2009). In the context of canonical autophagy, lipidated LC3 is conjugated to double-membrane autophagosomes where it is essential for cargo loading and autophagosome maturation. LC3- and STING-positive membranes in contrast are single-membrane vesicles that cluster at the perinuclear region (Fischer et al, 2020). LC3-lipidation onto single-membrane vesicles is generally dubbed as “conjugation of ATG8 (LC3) to single membranes” (CASM). How STING can drive CASM has not been clear (Fig. 1).

Two recent studies independently solved this intriguing question and revealed that STING is a proton channel (Xun et al, 2024; Liu et al, 2023). The authors show that STING activation de-acidifies the lumen of the Golgi and perinuclear endosomes using a Golgi pH sensor (Golgi-localized galactosyltransferase tagged with superecliptic pHluorin/mRuby) or an endosomal pH sensor (Lyso-pHluorin), respectively. A chemical agonist of STING, compound 53 (C53), previously shown to bind to the STING transmembrane domain (Lu et al, 2022), binds to and blocks its channel, inhibits STING-mediated proton efflux in vitro, STING-dependent organelle de-acidification, and CASM. Thus, both studies unequivocally reveal that CASM depends on proton efflux through the STING pore.

The activation of STING often leads to cell death and inflammasome activation that results in the processing and release of IL-1β. Intriguingly, C53 also inhibits these two processes but has no effect on STING-induced TBK1/interferon signaling. The classical function of STING in innate immune signaling (channel independent) can thus be uncoupled from its role in cell death and inflammasome activation (ion channel dependent).

On its route to lysosomes STING encounters a gradual acidification of the luminal pH of organelles along the secretory pathway, e.g., pH 7.2 (the ER), 6.7 (cis-Golgi), 6.0 (TGN), and 6.2 (REs) (Casey et al, 2010). The study by Xun and co-authors (Xun et al, 2024) details the target organelles of CASM and shows that TGN-derived perinuclear vesicles and REs are the primary targets of STING-induced LC3 lipidation. Given that the luminal pH of these organelles is mildly acidic, it is quite reasonable that STING-induced CASM occurs exclusively at these organelles, but not at ER and cis-Golgi where the luminal pH is neutral.

Very recent single-molecule imaging of STING reveals that STING becomes clustered at the TGN (about 20 STING molecules per cluster) (Kemmoku et al, 2024). The clustering requires STING palmitoylation and the Golgi lipid order defined by cholesterol. STING remains clustered at REs. Therefore, mass action of STING as cluster on TGN and REs may maximize the proton efflux, facilitating the de-acidification of luminal pH of these organelles.

Lysosomes directly encapsulate STING vesicles originating from REs and this process, named lysosomal microautophagy, is required for STING degradation and the termination of STING signaling (Kuchitsu et al, 2023). It has been unclear why cells use lysosomal microautophagy for STING degradation rather than delivery by membrane fusion between STING vesicles and lysosomes. The proton channel activity of STING provides an explanation for this choice. In the event of membrane fusion between STING vesicles and the lysosome, STING is transferred to the limiting membrane of lysosomes, which is expected to result in massive proton efflux from the lysosomal lumen with negative impacts on cell function and survival. Microautophagy, in contrast, delivers STING to the interior space of lysosomes so that it can be degraded without causing lysosomal damage.

CASM not only involves LC3 conjugation to phosphatidylethanolamine (PE), but also to phosphatidylserine (PS) (Durgan et al, 2021). The cytosolic leaflet of the RE membrane is enriched with PS (Hasegawa et al, 2021), which contributes to the retrograde trafficking from REs to the Golgi, recycling trafficking to the PM, and cell proliferation through the activation of YAP. These RE functions are mediated by a number of PS-binding effectors, such as evectin-2, EHD1, and PPP1R12A. STING-induced CASM might mask the polar head group of PS with LC3 and potentially interfere with the binding and function of these PS effectors, which would contribute to combat the invading pathogens.

STING-associated vasculopathy with onset in infancy (SAVI) is a disorder involving abnormal inflammation throughout the body, especially in the skin, blood vessels, and lungs. A number of STING variants (H72N, F153V, V147L, N154S, V155M, G158A, G166E, C206Y, G207E, R281Q/W, and R284G/S) have been identified in SAVI patients. Variant clinical manifestations have been described for SAVI patients. Because SAVI variants can form a homodimer (Shindo et al, 2022), it is tempting to speculate that the proton channel activities of individual SAVI dimers may be different and underlie the different clinical manifestations.

Overall, the two studies (Xun et al, 2024; Liu et al, 2023) bring new aspects into the pathophysiology of STING. Further research in this area may have important implications for understanding the pathogenesis of a number of STING-associated diseases and the development of targeted therapies.
